# Global trends and patterns in cardiovascular disease burden attributable to low physical activity: A systematic analysis for Global Burden of Disease Study from 1990 to 2021

**DOI:** 10.1371/journal.pone.0323374

**Published:** 2025-05-07

**Authors:** Rongxiang Zhang, Siyue Fan, Chenyang Zhu, Shiqi Chen, Feng Tian, Pingping Huang, Yuan Chen

**Affiliations:** 1 Xiamen Cardiovascular Hospital of Xiamen University, School of Medicine, Fujian Branch of National Clinical Research Center for Cardiovascular Diseases, Xiamen, China; 2 School of Nursing, Fujian University of Traditional Chinese Medicine, Fuzhou, China; National center for chronic and non-communicable diesease prevention and control, CHINA

## Abstract

**Background:**

This study analyzes the global burden of cardiovascular diseases (CVD) related to low physical activity from 1990 to 2021, focusing on spatiotemporal changes.

**Method:**

Using data from the GBD study, we examined trends in CVD burden linked to low physical activity, including mortality counts, disability-adjusted life years (DALYs), age-standardized mortality rates (ASMR), and age-standardized disability rates (ASDR). Decomposition analysis was used to identify key drivers of these changes, and frontier analysis visualized each country's potential to reduce the burden. An autoregressive integrated moving average model was used to forecast the burden from 2022 to 2036.

**Results:**

In 2021, approximately 370,000 deaths globally were attributed to CVD due to low physical activity. The ASMR and ASDR for CVD were 4.53 per 100,000 (95% uncertainty interval: 1.52 to 8.05) and 85.95 (95% UI: 35.25 to 140.65), respectively. From 1990 to 2021, the global burden increased, particularly in regions with a middle socio-demographic index, driven by aging populations and population growth. The ASMR is projected to decrease to 3.49 per 100,000 by 2036.

**Conclusions:**

Low physical activity is a major contributor to CVD-related mortality and disability worldwide. Public health interventions aimed at increasing physical activity, especially in regions with rising burdens, are essential to reduce the global CVD burden.

## Introduction

With the development of socioeconomic conditions and changing lifestyles, unhealthy habits have become more prevalent, increasing the impact of cardiovascular disease (CVD) risk factors on public health. CVD remains a leading cause of global mortality and disability [1,2]. A systematic analysis shows that the global number of CVD cases rose significantly from 31.31 million in 1990 to 55.45 million in 2019, a 77.12% increase. Similarly, CVD-related deaths surged from 12.07 million in 1990 to 18.56 million in 2019, reflecting a 53.81% rise [3]. CVD incidence varies across regions: in high-income countries, mortality rates have decreased due to advances in prevention and treatment. However, in many low- and middle-income countries, CVD burdens continue to rise, driven largely by aging populations and rapid urbanization [2,4,5]. This growing economic burden has become a major public health concern.

Low physical activity (LPA) is a modifiable risk factor for CVD. Studies have shown that regular physical activity can prevent CVD by lowering blood pressure, improving lipid profiles, and aiding weight management [6–8]. However, as urbanization and technological advancements reshape modern lifestyles, the global prevalence of LPA has increased, negatively impacting cardiovascular health [9,10]. It is estimated that LPA is associated with 7.2% of global all-cause mortality and 7.6% of CVD-specific mortality [11]. From 1990 to 2019, the number of CVD deaths linked to LPA increased significantly, reaching 639,170 deaths in 2019 [12].

Given the strong association between LPA and the CVD burden, effective public health interventions are urgently needed. Improving physical activity levels worldwide could significantly reduce CVD incidence and alleviate its economic burden [13]. This study, based on data from the 2021 Global Burden of Disease (GBD) Study, systematically analyzes the global CVD burden attributable to LPA, examines disparities across countries and regions, and provides scientific evidence to support policy development.

## Materials and methods

### Data sources

This study uses data from the GBD research, a comprehensive initiative to estimate the global burden of diseases. The data from 1990 to 2021 are available through the Global Health Data Exchange platform (http://ghdx.healthdata.org/gbd-results-tool), which provides standardized reports segmented by location, time, age, and sex. These reports follow WHO guidelines, ensuring transparency and accuracy in health estimates, including disease prevalence, incidence, mortality, and disability-adjusted life years (DALYs).

DALYs are calculated by combining years of life lost (YLL) due to premature mortality and years lived with disability (YLD) due to disease. This is done using life tables and estimates of disease prevalence and disability burden. Countries and regions are further categorized into 21 GBD regions and five Socio-demographic Index (SDI) groups: low, low-middle, middle, high-middle, and high SDI. The SDI is a composite measure of socio-economic development based on income, education, and fertility rates [12].

Detailed definitions of cardiovascular disease (CVD) have been provided in previous GBD reports [2]. To assess the impact of low physical activity (LPA) on global health, GBD 2021 utilized data from the Global Physical Activity Questionnaire, the International Physical Activity Questionnaire, and other sources. These tools collected information on the frequency, duration, and intensity of physical activity among adults aged 25 and older, across different countries, years, age groups, and sexes. Physical activity exposure was measured using metabolic equivalent tasks (METs), which represent the ratio of energy expenditure during activity relative to the basal metabolic rate. Physical activity levels were classified into four categories: inactive (<600 MET-minutes per week), low activity (600–3999 MET-minutes per week), moderate activity (4000–7999 MET-minutes per week), and high activity (≥8000 MET-minutes per week) [14].

### Statistical analysis

This study uses data on mortality, DALYs, YLL, (YLD, age-standardized mortality rates (ASMR), and age-standardized DALY rates (ASDR) to assess the burden of CVD attributable to LPA for comparison purposes.

To evaluate the temporal trends of disease burden, we applied the Joinpoint regression model, a set of linear statistical models [15,16]. This model identifies changes in disease incidence by calculating the least squares residuals, which avoids the subjectivity often seen in trend analyses based on linear assumptions. Turning points in the trends are identified by the sum of squared residuals between estimated and actual values. Joinpoint (version 5.2.0.0, April 2024; Statistical Research and Applications Branch, National Cancer Institute) was used for the analysis. We also calculated the average annual percentage change (AAPC) and tested whether fluctuations in different regions were statistically significant by comparing the AAPC to zero. A p-value of less than 0.05 was considered statistically significant.

Decomposition analysis was used to illustrate the contribution of three key factors—aging, population growth, and epidemiological changes—driving mortality changes from 1990 to 2021. Epidemiological changes refer to adjustments in mortality and incidence rates after controlling for age and population [17]. Frontier analysis was applied to assess the relationship between disease burden and socio-demographic development. This non-linear frontier, representing the minimal achievable burden based on development levels, was generated using non-parametric data envelopment analysis, as described in previous studies [18]. The efficiency gap, defined as the difference between a country's observed ASDR and the frontier, represents unrealized health gains based on the country's current development level.

To predict future trends, we used the AutoRegressive Integrated Moving Average (ARIMA) model, as in previous research [19], to analyze age-standardized mortality rates from 1990 to 2021 and forecast global trends and trends across five SDI regions for the next 15 years. ARIMA was chosen for its ability to model non-stationary time series by incorporating differencing, making it well-suited for long-term epidemiological forecasting. The ARIMA model includes autoregressive and moving average components, assuming that the data series is a time-varying random variable. Past values are used to predict future values. Differencing was applied to stabilize the time series data, and the auto.arima() function was used to select the optimal model based on the Akaike Information Criterion [19]. The AIC criterion was used to identify the best-fitting model while balancing complexity and predictive accuracy. Residual distribution normality was checked using Q-Q plots, autocorrelation function plots, and partial autocorrelation function plots. The Ljung-Box test was used to assess whether the residuals were white noise. ARIMA analysis and all plots were performed using R version 4.4.1. Statistical significance was set at a two-tailed p-value of less than 0.05.

## Results

### Global burden of CVD attributable to LPA in 2021

In 2021, the global burden of CVD attributable to LPA was substantial, affecting various indicators such as mortality, DALYs, YLDs, and YLLs. Global CVD mortality due to LPA was 371,736 cases (95% UI: 29,485–653,974), with an age-standardized mortality rate of 4.53 per 100,000 population (95% UI: 1.52 to 8.05). Of these, 141,434 were male and 230,302 were female. The global DALY burden was 7,294,918 years (95% UI: 3,040,412–11,863,376), with an age-standardized rate of 85.95 per 100,000 (95% UI: 35.25 to 140.65). Males accounted for 3,006,608 DALYs, while females accounted for 4,288,310 DALYs, indicating that women experience a greater overall disease burden due to CVD. The global YLD burden was 725,182 years (95% UI: 292,653–1,219,644), with an age-standardized rate of 8.37 per 100,000 (95% UI: 3.36 to 14.25). Males experienced 271,381 YLDs, while females had 453,801 YLDs. YLLs totaled 6,569,737 years (95% UI: 2,698,060–10,752,623), with an age-standardized rate of 77.57 per 100,000 (95% UI: 31.76 to 127.98). Males accounted for 2,735,228 YLLs, and females for 3,834,509 YLLs, indicating that women not only experience more years lost due to disability but also suffer greater premature mortality. For detailed data, refer to S1 Table.

The burden of CVD attributable to LPA varied significantly across different SDI regions in 2021. The middle SDI region had the highest burden, with 129,115 deaths and an age-standardized mortality rate of 5.56 per 100,000 (95% UI: 1.85 to 9.8). Total DALYs in this region amounted to 2,666,494 years (age-standardized rate of 104.39 per 100,000), which may be due to multiple factors, such as limited healthcare access, lower awareness of physical activity benefits, and a higher prevalence of other CVD risk factors, including poor diet and smoking. The low-middle SDI region followed, with 68,636 deaths and 1,521,849 DALYs (age-standardized rate of 111.53 per 100,000). Although the low-middle SDI region had fewer deaths than the middle SDI region, its higher age-standardized DALYs suggest a greater overall disease burden due to prolonged disability and inadequate disease management, potentially linked to economic and healthcare disparities.

The high SDI region had the lowest mortality, with 52,120 deaths (95% UI: 14,132–99,440) and an age-standardized mortality rate of 2.06 per 100,000 (95% UI: 0.64 to 3.83), which may be attributed to a combination of well-developed cardiovascular disease management, greater healthcare accessibility, higher levels of physical activity, and healthier lifestyle behaviors. The low SDI region reported 12,753 deaths (age-standardized rate of 3.29 per 100,000) and 303,280 DALYs (age-standardized rate of 64.94 per 100,000), indicating a still significant health impact in this region. For detailed data, refer to S1 Table.

### Temporal trends from 1990–2021

Trends in the global and SDI-specific burden of CVD attributable to LPA from 1990 to 2021 were analyzed using Joinpoint regression. The results revealed a global decline in both CVD mortality and DALYs, with AAPC of －1.26% for mortality and －1.12% for DALYs. The reduction was more significant in women (－1.40% for mortality and －1.25% for DALYs) compared to men (－0.87% for mortality and －0.83% for DALYs) (Table 1).

**Table 1:** Trends in Total Burden of Cardiovascular Disease Due to low physical activity from Joinpoint Regression, 1990 to 2021.

Measure	region	Both AAPC(95% CI)	Male AAPC(95% CI)	Female AAPC(95% CI)
Deaths	Global	-1.2605*(-1.4340 to -1.0866)	-0.8673*(-1.1105 to -0.6235)	-1.4018*(-1.5877 to -1.2156)
	High SDI	-3.4403*(-3.6803 to -3.1997)	-3.1998*(-3.3969 to -3.0024)	-3.5130*(-3.7758 to -3.2495)
	High-middle SDI	-1.2535*(-1.5889 to -0.9169)	-0.9275*(-1.2339 to -0.6201)	-1.3578*(-1.7022 to -1.0122)
	Middle SDI	-0.4022*(-0.6395 to -0.1643)	0.0528(-0.3700 to 0.4774)	-0.6595*(-0.8316 to -0.4871)
	Low-middle SDI	-0.1279(-0.4112 to 0.1562)	0.2354(-0.0731 to 0.5448)	-0.3807*(-0.7293 to -0.0308)
	Low SDI	-0.3147*(-0.6147 to -0.0137)	-0.0801(-0.4179 to 0.2588)	-0.5006*(-0.9888 to -0.0099)
DALYs	Global	-1.1223*(-1.2700 to -0.9744)	-0.8310*(-0.9566 to -0.7053)	-1.2452*(-1.4045 to -1.0856)
	High SDI	-2.9815*(-3.1664 to -2.7962)	-2.8260*(-2.9668 to -2.6851)	-3.0205*(-3.2259 to -2.8146)
	High-middle SDI	-1.2177*(-1.5159 to -0.9186)	-0.9092*(-1.1917 to -0.6260)	-1.3434*(-1.6644 to -1.0213)
	Middle SDI	-0.5166*(-0.7453 to -0.2874)	-0.1483(-0.4243 to 0.1285)	-0.7655*(-0.9361 to -0.5946)
	Low-middle SDI	-0.2827*(-0.5167 to -0.0481)	-0.0000(-0.2725 to 0.2732)	-0.5231*(-0.7457 to -0.2999)
	Low SDI	-0.4385*(-0.5907 to -0.2861)	-0.2318(-0.4750 to 0.0120)	-0.6250*(-0.9385 to -0.3105)

* Indicates that the AAPC is sianificantly different from zero at the alpha = 0.05 level

The largest declines were observed in high SDI regions, where mortality and DALYs decreased by 3.44% and 2.98%, respectively. In contrast, reductions were smaller in lower-income regions. Specifically, low-middle SDI and low SDI regions saw limited changes, with male populations in some areas even experiencing stagnation or slight increases in CVD burden, such as a 0.24% increase in mortality rates among men in low-middle SDI regions (Table 1).

### Age and sex patterns

**Fig 1:**
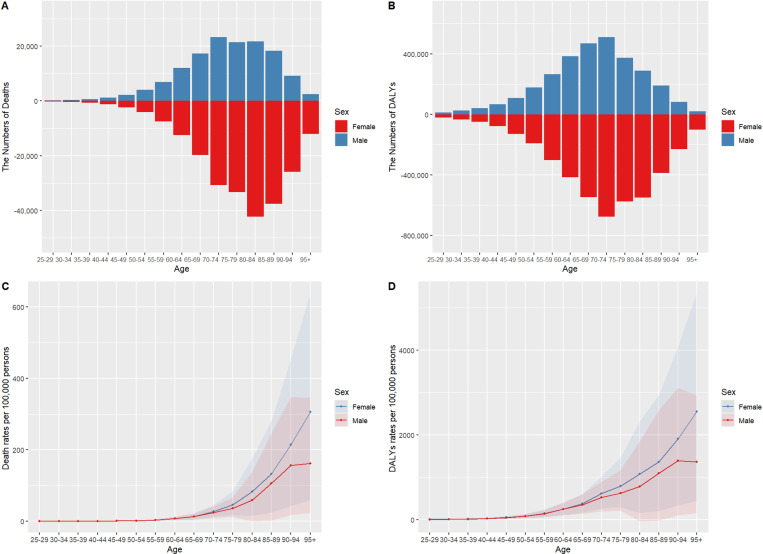
Global CVD Burden by Age and Sex (2021). Panel A shows the increase in mortality rates with age, particularly in males aged 60-74 and females aged 60-84. Panel B highlights DALY distribution, which increases significantly with age, especially for females post-55. Panel C shows higher mortality rates in females after age 60, while Panel D illustrates a more pronounced rise in DALYs for females. These findings underline the aging-related disparities in CVD burden, with a more substantial impact on females.

Fig 1A shows that in 2021, the global burden of CVD attributable to LPA increased with age in the 25–84 age group. This trend was particularly noticeable in individuals over 40, with a sharp rise in mortality rates among males aged 60–74 and females aged 60–84. Mortality rates for both sexes increased significantly after the age of 60 (Fig 1C), highlighting the elevated risk of CVD linked to LPA in this age group. Males consistently had lower mortality rates than females across all age groups.

Fig 1B illustrates the distribution of DALYs, which followed a similar pattern to mortality, showing a marked rise with age. Males had lower DALYs than females in all age categories, with the gap widening after age 55, indicating greater health losses among females due to LPA. These trends are further supported by the increases in both mortality (Fig 1C) and DALY rates (Fig 1D), which exhibit similar upward trajectories.

**Fig 2:**
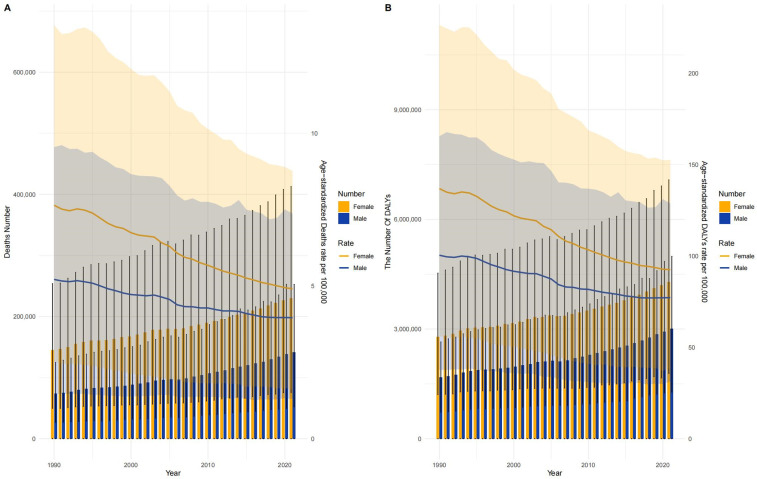
Global Mortality and DALYs for CVD by Sex (1990-2021). This figure displays global trends in CVD mortality rates and DALYs attributable to low physical activity (LPA) from 1990 to 2021. Both male and female mortality and DALY rates decreased annually, but total deaths and DALYs increased over time. Females experienced a consistently higher burden, with significantly higher age-standardized mortality rates and DALY values compared to males, emphasizing persistent gender disparities in CVD outcomes related to LPA.

From 1990 to 2021, global mortality rates and DALYs due to CVD attributable to LPA declined for both men and women. However, while the rates decreased annually, the total number of deaths and DALYs steadily increased, which may be attributed to global population growth and aging, leading to a larger at-risk population despite improvements in individual risk reduction. Notably, women consistently experienced a greater burden than men during this period. Specifically, female mortality numbers, age-standardized mortality rates (per 100,000 population), and DALY burdens were significantly higher than those for males in all years, with women experiencing disproportionately higher YLLs, indicating greater premature mortality. This trend reflects a persistent gender disparity in the CVD burden linked to LPA, with women disproportionately affected from 1990 to 2021. (Fig 2)

### Regional variations and SDI-specific analysis

**Fig 3:**
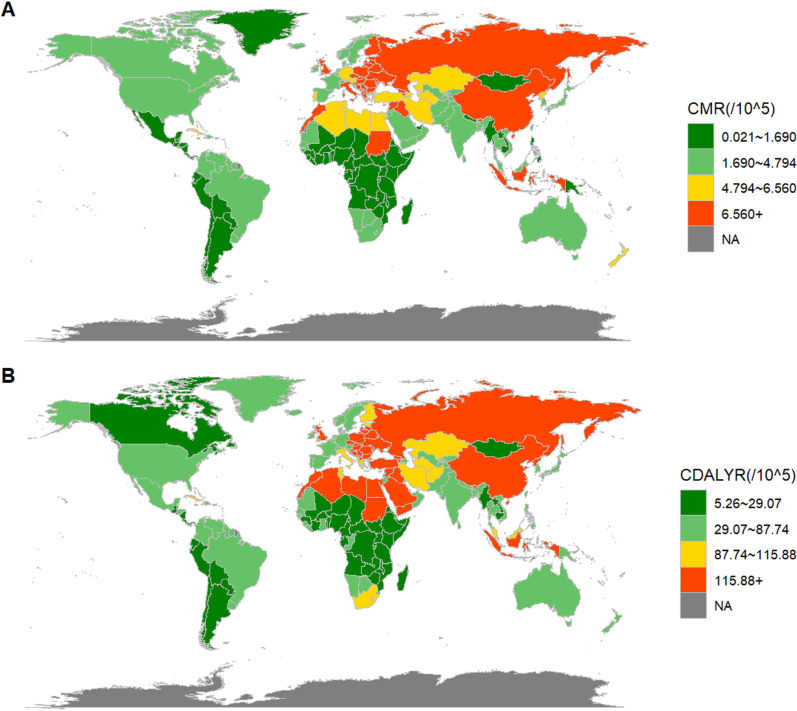
Burden of CVD due to physical inactivity in 204 countries in 2021. **(A)** Crude cardiovascular disease mortality rate (CMR) due to physical inactivity for each country in 2021; **(B)** Crude DALY rate for CVD due to physical inactivity for each country in 2021.CDALY, Crude Disability Adjusted Life Years. This map was created by the authors using R software (ggplot2, sf, and rnaturalearth packages) and public domain data from Natural Earth (https://www.naturalearthdata.com/).

The burden of CVD attributable to LPA varies significantly across countries and regions (Fig 3). In the figure, countries shaded in green have lower crude mortality and DALY rates, primarily in North America and much of Western Europe, especially in developed nations, indicating a lower health impact from LPA. In contrast, countries with medium burdens, shown in orange and light yellow, are mostly in South America, Southeast Asia, and parts of Africa, suggesting that cardiovascular health improvement is still needed in these regions. The red areas, representing higher crude mortality and DALY rates, are concentrated in certain African and Asian countries, highlighting the severe health impact of LPA in these regions due to limited healthcare resources and significant cardiovascular disease-related losses.

**Fig 4:**
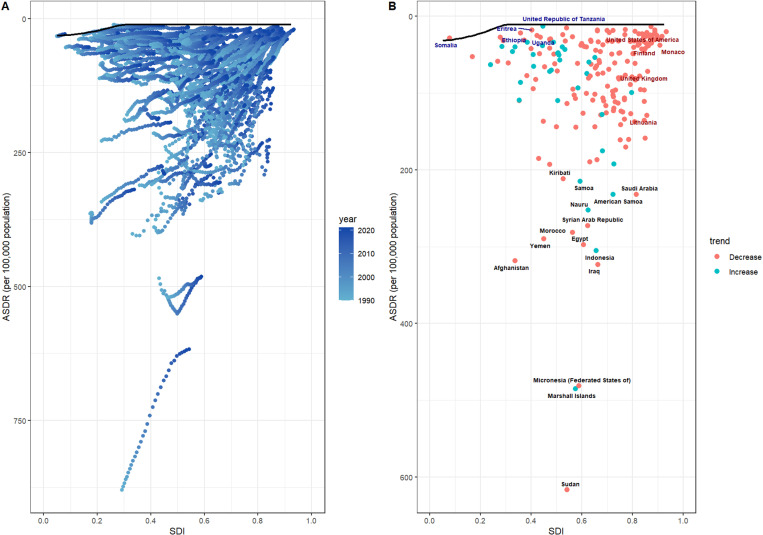
Trends in ASDR for CVD by SDI Level (1990-2021). Panel A shows a general decline in age-standardized death rates (ASDR) for CVD attributable to LPA, with the largest reductions seen in high-SDI countries. Panel B highlights that low-SDI countries, like Eritrea, saw an increase in ASDR, while high-SDI nations such as the **U.**S. and Finland showed significant reductions. War-torn countries such as Afghanistan and Syria still report high ASDR, despite slight improvements, reflecting the relationship between socioeconomic development and CVD burden.

Trends in age-standardized ASDR for CVD attributable to LPA across different SDI countries show a general improvement over time (Fig 4A). By 2021, ASDR had notably decreased compared to 1990, especially in high-SDI countries and regions (Fig 4A). This trend was consistent over the years, although the extent of reduction varied depending on the SDI level of the country. The data (Fig 4B) show that low-SDI countries, such as Eritrea, have experienced an increase in cardiovascular disease burden, while high-SDI countries like the U.S., Finland, and the U.K. have seen a significant reduction. This indicates a strong link between a country's socioeconomic development and changes in cardiovascular disease burden. However, countries affected by war or instability, such as Afghanistan, Syria, and Yemen, continue to have high ASDR, despite having medium SDI levels, although a slight decline in burden has been observed.

### Decomposition analysis of Deaths rates

**Fig 5:**
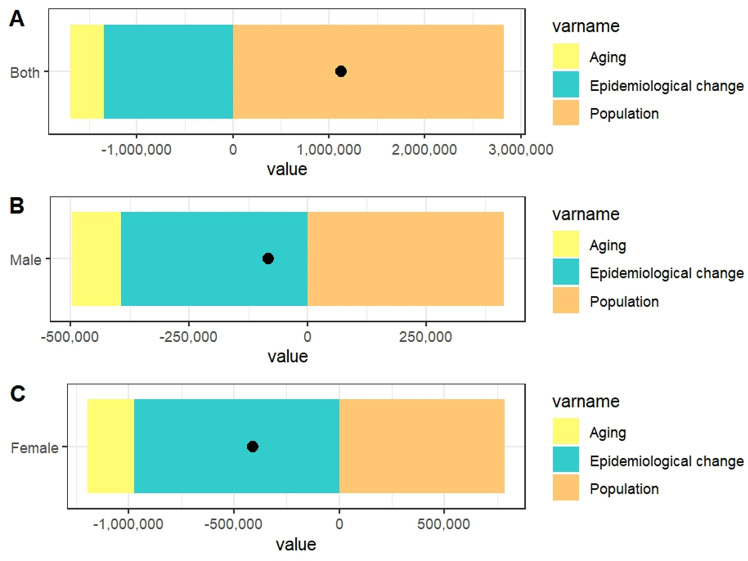
Impact of Aging, Epidemiological Shifts, and Population Growth on CVD Burden (1990-2021). This figure illustrates the contributions of aging, epidemiological shifts, and population growth to global disparities in CVD burden over the past 30 years. Aging contributed the most to negative differences (56.36%), especially in males. Epidemiological shifts had the largest negative effect, particularly in males (－472.35%) and females (－237.53%). Population growth mitigated these effects, especially in males (－496.9%) and somewhat in females. These findings emphasize the distinct impacts of demographic factors on the global CVD burden.

Over the past 30 years, global disparities in the CVD burden have been influenced by aging, epidemiological shifts, and population growth (Fig 5, S2 Table). Aging and epidemiological changes contributed 56.36% and 237.08% to the overall negative differences, respectively, with aging having the greatest impact on males (124.55%) and epidemiological shifts causing the most significant negative effect on males (－472.35%). In contrast, population growth helped mitigate the burden, contributing －193.44% to the overall difference, with a more positive impact on females (－191.51%). Notably, epidemiological shifts had a negative effect across all groups, particularly on females (－237.53%). Population growth alleviated negative trends, especially in males, reducing the burden by －496.9%, and had a more pronounced positive impact on females. These findings highlight the distinct impacts of aging, epidemiology, and population dynamics on different sex groups, emphasizing the need for targeted policy interventions.

### Future projections

**Fig 6:**
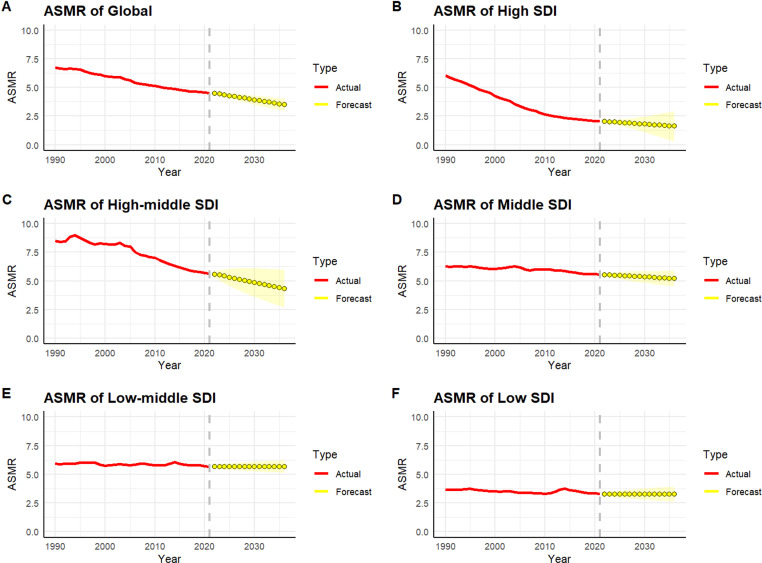
Projected ASMR for CVD and LPA (2022-2036). This figure shows projected trends in age-standardized mortality rates (ASMR) for cardiovascular diseases attributable to low physical activity (LPA) from 2022 to 2036, based on ARIMA modeling. Global ASMR is expected to decline from 4.48% in 2022 to 3.49% in 2036, with the most significant reductions in high-SDI regions. In contrast, low-SDI regions will experience slower declines. These trends highlight the global efforts to reduce CVD mortality, while disparities remain, particularly in lower-income regions.

We used the ARIMA model to predict trends in ASMR due to LPA and cardiovascular diseases over the next 15 years. The optimal ARIMA model, selected using the auto.arima() function, showed a good fit, as confirmed by Q-Q plots, ACF, PACF, and the Ljung-Box test, indicating that the residuals followed white noise characteristics (S1 Fig. and S3 Table). The results (Fig 6) show a global decline in ASMR for cardiovascular diseases since 1990, with a continued decrease expected, from 4.48% in 2022 to 3.49% in 2036. High SDI regions are projected to experience the most significant decrease, from 2.03% in 2022 to 1.61% by 2036. In contrast, low SDI and lower-middle SDI regions are expected to see more limited declines, reflecting disparities in disease burden management. The ASMR in upper-middle and middle SDI regions is expected to decline gradually over the same period.

## Discussion

This study conducted a systematic analysis of the temporal trends in the global burden of CVD specifically attributable to LPA from 1990 to 2021, stratified by sex, region, and SDI. The findings show a general decline in both mortality rate and DALY rate associated with LPA-induced CVD globally between 1990 and 2021. However, the absolute numbers of deaths and DALYs have been rising annually [12,20]. Notably, middle SDI regions currently bear the highest CVD burden. The primary drivers of the increase in CVD-related mortality due to LPA during this period are aging populations and epidemiological transitions. It is anticipated that this mortality rate will continue to decline from 2022 to 2036.

The health burden from CVD attributable to LPA has remained disproportionately higher in women, which contrasts with some previous studies that reported a higher CVD burden among men [12]. This distinctive finding may be attributed to several factors, including the use of the updated GBD 2021 database with improved methodologies and the extended study period (1990–2021), which captures the impact of the COVID-19 pandemic. From a biological perspective, women's distinct physiological makeup and hormone levels play a crucial role in influencing CVD risk, particularly during the post-menopausal period [21]. Social determinants also show marked sex differences, with women facing unique barriers to physical activity, such as safety concerns, cultural restrictions, and competing family-work responsibilities, which may have been exacerbated by the pandemic. This may reflect true sex differences in how physical inactivity impacts cardiovascular health, with women bearing a higher CVD burden than men due to insufficient physical activity. It would be valuable for future research to explore the potential biological, social, and behavioral explanations for these disparities. To address these sex-specific disparities, interventions should focus on developing gender-sensitive physical activity programs that consider both biological factors and sociocultural barriers, while future research should further investigate these sex differences using standardized methodologies [9].

Between 1990 and 2021, substantial disparities in the CVD burden attributable to LPA were observed across nations and regions. These disparities were largely driven by varying levels of economic development [22]. Low-SDI countries, including many in Africa, consistently exhibited higher ASMR, while higher-SDI countries, like those in North America and Europe, generally showed lower ASMR, indicating a strong inverse relationship between economic status and cardiovascular health. High-burden countries were predominantly in low-SDI regions, highlighting deficits in healthcare infrastructure and resource availability. Conversely, higher-SDI nations showed significant progress in reducing CVD mortality rates. Nonetheless, deaths from armed conflict and terrorism remain a critical global health concern [23]. Even in countries with moderate SDI levels, those experiencing war or instability continue to display high CVD burdens, despite a general decline in mortality rates. Our findings suggest that in economically diverse settings, particularly low- and middle-income nations, higher levels of physical inactivity are associated with increased risks of all-cause mortality and cardiovascular disease. Encouraging regular physical activity through population-level interventions has the potential to be a beneficial approach for mitigating the CVD burden [24–26]. And, Effective interventions could include implementing community-based exercise programs, integrating physical activity promotion into primary healthcare services, developing safe and accessible walking and cycling infrastructure, enhancing school-based physical education, and launching workplace wellness initiatives. These strategies can help foster long-term behavioral changes and reduce physical inactivity at a population level.

The association between LPA and CVD burden becomes particularly evident in older populations, with our analysis showing that adults aged 60 and above represent a substantial portion of this burden [27]. Age-related declines in physical function and the accumulation of chronic conditions significantly increase the risk of CVD in older adults [28]. Research indicates that older adults who maintain insufficient levels of physical activity face elevated risks of adverse cardiovascular outcomes [29]. Demographic shifts over the past 32 years have positively impacted CVD burdens linked to LPA, particularly in high-SDI regions, where the burden has decreased [30]. These areas benefit from advanced healthcare systems, comprehensive public health policies, and healthier lifestyle behaviors driven by economic development.

This study has several limitations. First, distinguishing between deaths caused by CVD and those from comorbidities is challenging, which may lead to an underestimation of CVD-related mortality. Second, the data used in the study rely heavily on the GBD database, and data availability and quality vary across regions. Only a few countries and regions provide actual data, while many rely on modeled estimates or extrapolations from other areas. Third, the observed differences in CVD burden may reflect both real changes in disease patterns and methodological differences in attribution analysis and age-standardization procedures. Previous studies have also acknowledged these limitations [31,32]. Additionally, despite using various statistical methods to adjust the data, potential biases in the estimates remain unavoidable. Furthermore, although the latest GBD 2021 database provides updated methodologies and revised historical data, the impact of recent global events, particularly the COVID-19 pandemic, on data collection and quality needs careful consideration. Finally, the current estimates are based on past trends and covariates, which may contribute to a lag in GBD data updates [18]. Future studies should focus on harmonizing research methodologies across different time periods and investigating the complex interactions between sex, physical activity, and cardiovascular health outcomes.

## Conclusions

This study systematically assessed the global burden of CVD attributable to LPA and compared data across 204 countries and regions. The findings highlight significant disparities in the impact of LPA on CVD burden over the past 32 years. While mortality and DALY rates have generally declined, the absolute numbers of deaths and DALYs have continued to rise. The burden varied widely across countries and regions, with the highest impact observed in nations with medium SDI. Older adults and women experienced greater health losses.

## Supporting information

S1 Fig Q-Q Plots, ACF, and PACF of Residuals for Different SDI Countries. (TIF)

S1 Table Overview of Cardiovascular Disease Burden Due to Physical Insufficiency in Global and 5SDI Zones in 2021. (DOCX)

S2 Table Changes in Deaths rate according to population-level determinants and causes from 1990 to 2021. (DOCX)

S3 Table ARIMA Models and Ljung-Box Test Results for Cardiovascular Disease Burden in Different SDI Countries. (DOCX)

## References

[1] GBD 2021 Causes of Death Collaborators. Global burden of 288 causes of death and life expectancy decomposition in 204 countries and territories and 811 subnational locations, 1990-2021: a systematic analysis for the Global Burden of Disease Study 2021. Lancet. 2024;403(10440):2100–32. doi: 10.1016/S0140-6736(24)00367-2 38582094 PMC11126520

[2] Roth GA, Mensah GA, Johnson CO, Addolorato G, Ammirati E, Baddour LM, et al. Global Burden of Cardiovascular Diseases and Risk Factors, 1990-2019: Update From the GBD 2019 Study. J Am Coll Cardiol. 2020;76(25):2982–3021. doi: 10.1016/j.jacc.2020.11.010 33309175 PMC7755038

[3] Li Y, Cao G-Y, Jing W-Z, Liu J, Liu M. Global trends and regional differences in incidence and mortality of cardiovascular disease, 1990-2019: findings from 2019 global burden of disease study. Eur J Prev Cardiol. 2023;30(3):276–86. doi: 10.1093/eurjpc/zwac285 36458973

[4] Jagannathan R, Patel SA, Ali MK, Narayan KMV. Global Updates on Cardiovascular Disease Mortality Trends and Attribution of Traditional Risk Factors. Curr Diab Rep. 2019;19(7):44. 10.1007/s11892-019-1161-2 31222515 31222515

[5] Yusuf S, Joseph P, Rangarajan S, Islam S, Mente A, Hystad P, et al. Modifiable risk factors, cardiovascular disease, and mortality in 155 722 individuals from 21 high-income, middle-income, and low-income countries (PURE): a prospective cohort study. Lancet. 2020;395(10226):795–808. doi: 10.1016/S0140-6736(19)32008-2 31492503 PMC8006904

[6] Wahid A, Manek N, Nichols M, Kelly P, Foster C, Webster P, et al. Quantifying the Association Between Physical Activity and Cardiovascular Disease and Diabetes: A Systematic Review and Meta-Analysis. J Am Heart Assoc. 2016;5(9):e002495. doi: 10.1161/JAHA.115.002495 27628572 PMC5079002

[7] Shailendra P, Baldock KL, Li LSK, Bennie JA, Boyle T. Resistance Training and Mortality Risk: A Systematic Review and Meta-Analysis. Am J Prev Med. 2022;63(2):277–85. doi: 10.1016/j.amepre.2022.03.020 35599175

[8] Baffour-Awuah B, Pearson MJ, Dieberg G, Smart NA. Isometric Resistance Training to Manage Hypertension: Systematic Review and Meta-analysis. Curr Hypertens Rep. 2023;25: 35–49. doi: 10.1007/s11906-023-01232-w 36853479 PMC10014822

[9] Chomistek AK, Manson JE, Stefanick ML, Lu B, Sands-Lincoln M, Going SB, et al. Relationship of sedentary behavior and physical activity to incident cardiovascular disease: results from the Women’s Health Initiative. J Am Coll Cardiol. 2013;61(23):2346–54. doi: 10.1016/j.jacc.2013.03.031 23583242 PMC3676694

[10] Cunningham C, O’ Sullivan R, Caserotti P, Tully MA. Consequences of physical inactivity in older adults: A systematic review of reviews and meta-analyses. Scand J Med Sci Sports. 2020;30(5):816–27. doi: 10.1111/sms.13616 32020713

[11] Katzmarzyk PT, Friedenreich C, Shiroma EJ, Lee I-M. Physical inactivity and non-communicable disease burden in low-income, middle-income and high-income countries. Br J Sports Med. 2022;56:101–6. doi: 10.1136/bjsports-2020-103640 33782046 PMC8478970

[12] Zhang J, Yuan Z, Mo C, Kang Y, Wang F, Wei X, et al. The global burden of cardiovascular diseases and type 2 diabetes attributable to low physical activity, 1990-2019: an analysis from the global burden of disease study. Front Cardiovasc Med. 2023;10:1247705. doi: 10.3389/fcvm.2023.1247705 38173813 PMC10762785

[13] Dempsey PC, Rowlands AV, Strain T, Zaccardi F, Dawkins N, Razieh C, et al. Physical activity volume, intensity, and incident cardiovascular disease. Eur Heart J. 2022;43: 4789–4800. doi: 10.1093/eurheartj/ehac613 36302445

[14] Kyu HH, Bachman VF, Alexander LT, Mumford JE, Afshin A, Estep K, et al. Physical activity and risk of breast cancer, colon cancer, diabetes, ischemic heart disease, and ischemic stroke events: systematic review and dose-response meta-analysis for the Global Burden of Disease Study 2013. BMJ. 2016;354:i3857. https://doi.org/10.1136/bmj.i3857 PMID: 27510511 10.1136/bmj.i3857 PMC4979358 27510511

[15] Zhang Y, Liu J, Han X, Jiang H, Zhang L, Hu J, et al. Long-term trends in the burden of inflammatory bowel disease in China over three decades: A joinpoint regression and age-period-cohort analysis based on GBD 2019. Front Public Health. 2022;10: 994619. doi: 10.3389/fpubh.2022.994619 36159285 PMC9490087

[16] Wu S, Xu W, Guan C, Lv M, Jiang S, Jinhua Z. Global burden of cardiovascular disease attributable to metabolic risk factors, 1990-2019: an analysis of observational data from a 2019 Global Burden of Disease study. BMJ Open. 2023;13(5):e069397. doi: 10.1136/bmjopen-2022-069397 37173115 PMC10186407

[17] Xie Y, Bowe B, Mokdad AH, Xian H, Yan Y, Li T, et al. Analysis of the Global Burden of Disease study highlights the global, regional, and national trends of chronic kidney disease epidemiology from 1990 to 2016. Kidney Int. 2018;94: 567–81. doi: 10.1016/j.kint.2018.04.011 30078514

[18] Pan H, Zhao Z, Deng Y, Zheng Z, Huang Y, Huang S, et al. The global, regional, and national early-onset colorectal cancer burden and trends from 1990 to 2019: results from the Global Burden of Disease Study 2019. BMC Public Health. 2022;22(1):1896. doi: 10.1186/s12889-022-14274-7 36221047 PMC9555189

[19] Li Y, Ning Y, Shen B, Shi Y, Song N, Fang Y, et al. Temporal trends in prevalence and mortality for chronic kidney disease in China from 1990 to 2019: an analysis of the Global Burden of Disease Study 2019. Clin Kidney J. 2023;16: 312–321. doi: 10.1093/ckj/sfac218 36755850 PMC9900593

[20] Liu J, Liu Y, Ma W, Liu J, Tong Y, Wang C, et al. Age-period-cohort analysis of ischemic stroke deaths attributable to physical inactivity in different income regions. Sci Rep. 2024;14(1):6547. doi: 10.1038/s41598-024-57309-2 38503900 PMC10951293

[21] Gersh F, O’Keefe JH, Elagizi A, Lavie CJ, Laukkanen JA. Estrogen and cardiovascular disease. Prog Cardiovasc Dis. 2024;84:60–7. doi: 10.1016/j.pcad.2024.01.015 38272338

[22] Ding Q, Liu S, Yao Y, Liu H, Cai T, Han L. Global, Regional, and National Burden of Ischemic Stroke, 1990-2019. Neurology. 2022;98: e279–e290. doi: 10.1212/WNL.0000000000013115 34911748

[23] GBD 2017 Causes of Death Collaborators. Global, regional, and national age-sex-specific mortality for 282 causes of death in 195 countries and territories, 1980-2017: a systematic analysis for the Global Burden of Disease Study 2017. Lancet Lond Engl. 2018;392: 1736–1788. doi: 10.1016/S0140-6736(18)32203-7 PMC6227606 30496103

[24] Perry AS, Dooley EE, Master H, Spartano NL, Brittain EL, Pettee Gabriel K. Physical Activity Over the Lifecourse and Cardiovascular Disease. Circ Res. 2023;132: 1725–1740. doi: 10.1161/CIRCRESAHA.123.322121 37289900 PMC10254078

[25] Li S, Lear SA, Rangarajan S, Hu B, Yin L, Bangdiwala SI, et al. Association of Sitting Time With Mortality and Cardiovascular Events in High-Income, Middle-Income, and Low-Income Countries. JAMA Cardiol. 2022;7(8):796–807. doi: 10.1001/jamacardio.2022.1581 35704349 PMC9201743

[26] Lavie CJ, Ozemek C, Carbone S, Katzmarzyk PT, Blair SN. Sedentary Behavior, Exercise, and Cardiovascular Health. Circ Res. 2019;124: 799–815. doi: 10.1161/CIRCRESAHA.118.312669 30817262

[27] Qu C, Liao S, Zhang J, Cao H, Zhang H, Zhang N, et al. Burden of cardiovascular disease among elderly: based on the Global Burden of Disease Study 2019. Eur Heart J Qual Care Clin Outcomes. 2024;10: 143–153. doi: 10.1093/ehjqcco/qcad033 37296238 PMC10904724

[28] Ijaz N, Buta B, Xue Q-L, Mohess DT, Bushan A, Tran H, et al. Interventions for Frailty Among Older Adults With Cardiovascular Disease: JACC State-of-the-Art Review. J Am Coll Cardiol. 2022;79: 482–503. doi: 10.1016/j.jacc.2021.11.029 35115105 PMC8852369

[29] Bellettiere J, LaMonte MJ, Evenson KR, Rillamas-Sun E, Kerr J, Lee I-M, et al. Sedentary behavior and cardiovascular disease in older women: The Objective Physical Activity and Cardiovascular Health (OPACH) Study. Circulation. 2019;139: 1036–46. 31031411 10.1161/CIRCULATIONAHA.118.035312 PMC6481298

[30] Roth GA, Johnson C, Abajobir A, Abd-Allah F, Abera SF, Abyu G, et al. Global, Regional, and National Burden of Cardiovascular Diseases for 10 Causes, 1990 to 2015. J Am Coll Cardiol. 2017;70: 1–25. doi: 10.1016/j.jacc.2017.04.052 28527533 10.1016/j.jacc.2017.04.052 PMC5491406

[31] Hong L, Yan MM, Zhang YQ, Wang K, Wang YQ, Luo SQ, et al. Global Burden of Cardiovascular Disease Attributable to High Temperature in 204 Countries and Territories from 1990 to 2019. Biomed Environ Sci. 2023;36(3):222–30. doi: 10.3967/bes2023.025 37005076

[32] GBD 2019 Blindness and Vision Impairment Collaborators, Vision Loss Expert Group of the Global Burden of Disease Study. Trends in prevalence of blindness and distance and near vision impairment over 30 years: an analysis for the Global Burden of Disease Study. Lancet Glob Health. 2021;9: e130–e143. doi: 10.1016/S2214-109X(20)30425-333275950

