# Supplementary figures and images for: Global trends and patterns in cardiovascular disease burden attributable to low physical activity: A systematic analysis for Global Burden of Disease Study from 1990 to 2021
Source: PLoS One. 2025 May 7;20(5):e0323374. doi: 10.1371/journal.pone.0323374 (PMC12057944; doi:10.1371/journal.pone.0323374)

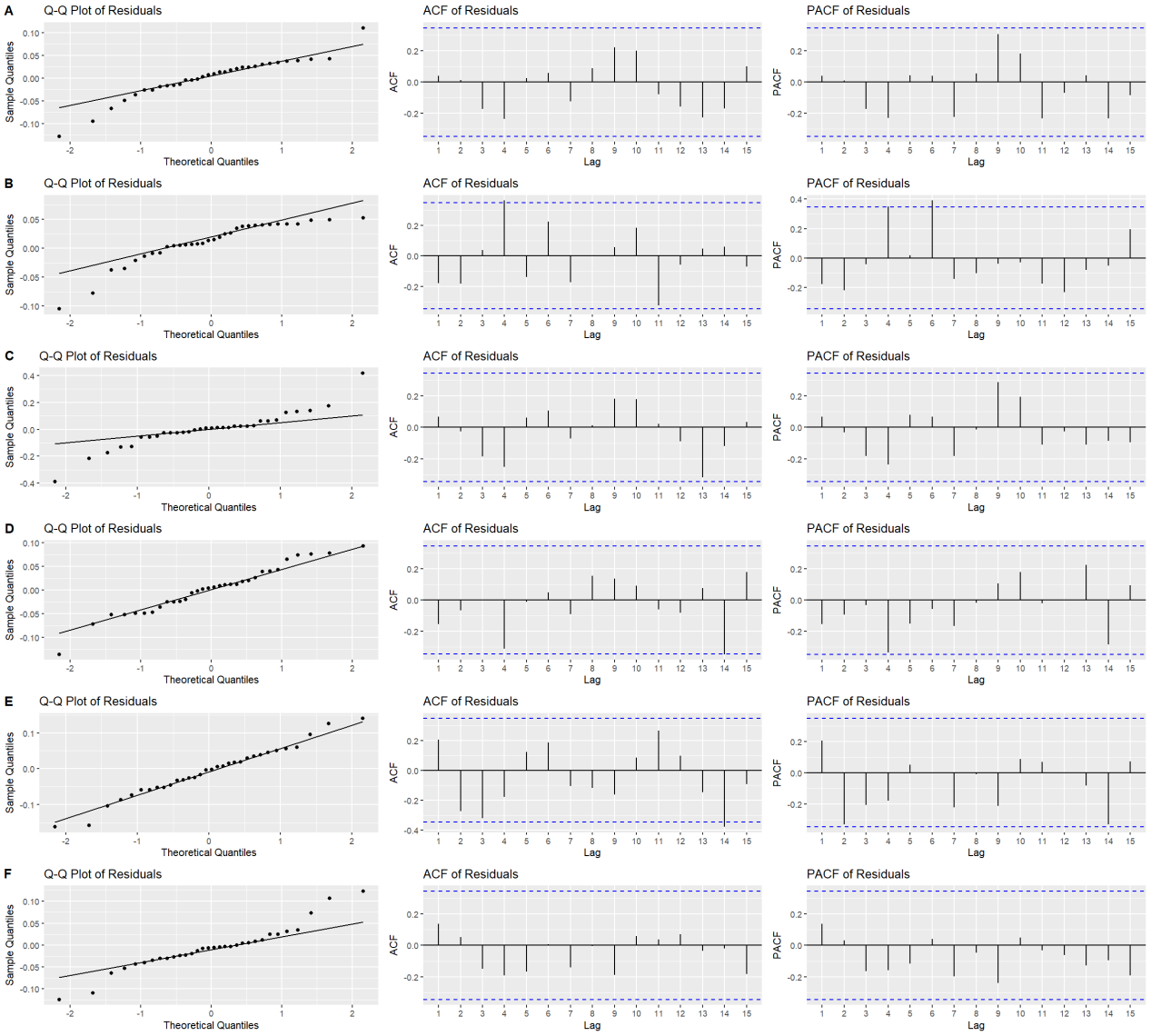

Supplement: S1 Fig — (TIF) [file pone.0323374.s001.tif]
