# Supplementary material for: Global trends and patterns in cardiovascular disease burden attributable to low physical activity: A systematic analysis for Global Burden of Disease Study from 1990 to 2021
Source: PLoS One. 2025 May 7;20(5):e0323374. doi: 10.1371/journal.pone.0323374 (PMC12057944; doi:10.1371/journal.pone.0323374)
Supplement: S1 Table — (DOCX) [file pone.0323374.s002.docx]

**S1 Table.** Overview of Cardiovascular Disease Burden Due to Physical Insufficiency in Global and 5SDI Zones in 2021.

| Measure | Location | All ages cases(95%UI) | | | Age standardized rates per 100,000 population (95%UI) | | |
| --- | --- | --- | --- | --- | --- | --- | --- |
|  |  | Total | Male | Female | Total | Male | Female |
| Deaths | Global | 371736(129485,653974) | 141434(52403,252555) | 230302(75807,412846) | 4.53(1.52,8.05) | 3.96(1.3,7.37) | 4.91(1.62,8.77) |
|  | High SDI | 52120(14132,99440) | 18291(5374,34152) | 33829(9036,66554) | 2.06(0.64,3.83) | 1.86(0.57,3.45) | 2.14(0.67,4.06) |
|  | High-middle SDI | 108720(34162,202854) | 37521(11731,73491) | 71198(21602,135646) | 5.65(1.74,10.62) | 4.83(1.35,9.78) | 6.08(1.88,11.46) |
|  | Middle SDI | 129115(51242,219952) | 54693(21142,99831) | 74422(29733,132422) | 5.56(1.85,9.8) | 5.24(1.6,9.98) | 5.78(1.99,10.45) |
|  | Low-middle SDI | 68636(26771,115298) | 25925(9949,43913) | 42711(16629,71558) | 5.67(2.02,9.87) | 4.54(1.59,7.99) | 6.61(2.46,11.3) |
|  | Low SDI | 12753(4916,21815) | 4879(1900,8375) | 7875(3073,13585) | 3.29(1.02,5.91) | 2.59(0.76,4.79) | 3.91(1.26,7.18) |
| DALYs | Global | 7294918(3040412,11863376) | 3006608(1274556,4986913) | 4288310(1785581,7080572) | 85.95(35.25,140.65) | 77.2(31.2,128.66) | 92.49(38.61,152.58) |
|  | High SDI | 886361(314423,1565542) | 365603(136111,636865) | 520757(168186,945785) | 40.7(16.23,69.35) | 39.14(15.65,67.06) | 41.13(16.53,71.59) |
|  | High-middle SDI | 1909651(758443,3346253) | 741482(301170,1298106) | 1168169(449142,2080254) | 97.21(38.29,171.02) | 87.13(33.65,158.58) | 103.18(40.66,181.7) |
|  | Middle SDI | 2666494(1172827,4318310) | 1181811(517802,1949933) | 1484682(624859,2414767) | 104.39(43.95,170.88) | 98.89(39.5,170.28) | 108.65(45.41,179.64) |
|  | Low-middle SDI | 1521849(693849,2393740) | 597322(256999,943653) | 924528(417046,1476033) | 111.53(47.46,180.69) | 91.2(35.85,151.29) | 129.29(55.96,209.99) |
|  | Low SDI | 303280(137069,480008) | 117780(50156,190533) | 185500(81536,298887) | 64.94(27.01,108.35) | 51.53(20.42,88.15) | 77.29(31.69,131.35) |
| YLDs | Global | 725182(292653,1219644) | 271381(108293,462050) | 453801(186134,764924) | 8.37(3.36,14.25) | 6.64(2.44,11.59) | 9.99(4.14,16.75) |
|  | High SDI | 148512(48607,267134) | 61909(22587,110160) | 86603(27853,156731) | 7.89(3.22,13.29) | 6.92(2.79,11.97) | 8.84(3.76,14.65) |
|  | High-middle SDI | 194164(74188,334580) | 71822(26456,125847) | 122342(47336,208217) | 9.91(3.84,17.04) | 7.93(2.72,14.25) | 11.74(4.84,19.73) |
|  | Middle SDI | 253851(106994,422610) | 92625(37211,157002) | 161226(68426,270015) | 9.27(3.72,15.87) | 7.11(2.68,12.58) | 11.29(4.7,19.19) |
|  | Low-middle SDI | 101675(45385,166965) | 36203(15279,60139) | 65472(29924,105797) | 6.7(2.88,11.14) | 5.04(2.06,8.5) | 8.26(3.69,13.58) |
|  | Low SDI | 26353(12050,41599) | 8606(3758,13618) | 17746(8299,28226) | 4.6(1.92,7.74) | 3.17(1.29,5.4) | 5.97(2.57,9.88) |
| YLLs | Global | 6569737(2698060,10752623) | 2735228(1183163,4487196) | 3834509(1566327,6450223) | 77.57(31.76,127.98) | 70.56(28.4,118.1) | 82.5(33.79,138.36) |
|  | High SDI | 737848(258728,1298902) | 303694(111958,525758) | 434154(141780,799567) | 32.81(12.72,55.38) | 32.22(12.65,55.14) | 32.29(11.82,56.22) |
|  | High-middle SDI | 1715486(669946,3006110) | 669660(265085,1180415) | 1045827(391179,1858851) | 87.3(33.67,153.89) | 79.2(30.6,145.71) | 91.44(35.64,161.01) |
|  | Middle SDI | 2412642(1039357,3910287) | 1089186(474008,1807522) | 1323456(546014,2148607) | 95.12(39.7,155.58) | 91.77(36.62,160.02) | 97.35(39.94,161.07) |
|  | Low-middle SDI | 1420175(642331,2236283) | 561119(238975,888626) | 859056(379889,1372065) | 104.84(44.06,170.36) | 86.17(33.84,143.05) | 121.02(51.64,197.54) |
|  | Low SDI | 276928(122726,441694) | 109174(45920,177703) | 167754(70756,272193) | 60.34(24.6,100.94) | 48.36(19.02,83.39) | 71.31(28.57,121.5) |
