# Supplementary material for: Global trends and patterns in cardiovascular disease burden attributable to low physical activity: A systematic analysis for Global Burden of Disease Study from 1990 to 2021
Source: PLoS One. 2025 May 7;20(5):e0323374. doi: 10.1371/journal.pone.0323374 (PMC12057944; doi:10.1371/journal.pone.0323374)
Supplement: S2 Table — (DOCX) [file pone.0323374.s003.docx]

**S2 Table.** Changes in Deaths rate according to population-level determinants and causes from 1990 to 2021.

| Location | Overll difference ^a^ | Change due to Population-level determinants (% contribute to the total changes) | | |
| --- | --- | --- | --- | --- |
|  |  | Aging ^b^ | Population ^c^ | Epidemiological change ^d^ |
| Global | -711200.4 | -400832.4 (56.36%) | 1375733 (-193.44%) | -1686101 (237.08%) |
| **Sex** |  |  |  |  |
| Female | -409928.2 | -221301.4 (53.99%) | 785057.5 (-191.51%) | -973684.3 (237.53%) |
| Male | -83401.3 | -103874.3 (124.55%) | 414419.3 (-496.9%) | -393946.3 (472.35%) |

a.Change in Deaths rate between year 2021 and 1990

b.Change in Deaths rate due to change in the age structure

c.Change in Deaths rate due to change in population number

d.Change in Deaths rate due to epidemiologic changes
