# Supplementary material for: Global trends and patterns in cardiovascular disease burden attributable to low physical activity: A systematic analysis for Global Burden of Disease Study from 1990 to 2021
Source: PLoS One. 2025 May 7;20(5):e0323374. doi: 10.1371/journal.pone.0323374 (PMC12057944; doi:10.1371/journal.pone.0323374)
Supplement: S3 Table — (DOCX) [file pone.0323374.s004.docx]

**S3 Table.** ARIMA Models and Ljung-Box Test Results for Cardiovascular Disease Burden in Different SDI Countries

| Location | ARIMA Model(p,d,q) | Ljung-Box Test | AIC |
| --- | --- | --- | --- |
| Global | (1,1,0) | X-squared = 8.7957  p-value = 0.5516 | -98.97 |
| High SDI | (0,2,1) | X-squared = 13.525  p-value = 0.1958 | -106.08 |
| High-middle SDI | (1,1,0) | X-squared = 8.0362  p-value = 0.6253 | -30.99 |
| Middle SDI | (0,1,1) | X-squared = 7.6181  p-value = 0.6661 | -91.18 |
| Low-middle SDI | (0,1,0) | X-squared = 14.074  p-value = 0.1697 | -74.37 |
| Low SDI | (0,1,1) | X-squared = 6.9382  p-value = 0.7313 | -92.47 |

AIC, Akaike Information Criterion.
